# Mutation analysis of the phospholamban gene in 315 South Africans with dilated, hypertrophic, peripartum and arrhythmogenic right ventricular cardiomyopathies

**DOI:** 10.1038/srep22235

**Published:** 2016-02-26

**Authors:** Maryam Fish, Gasnat Shaboodien, Sarah Kraus, Karen Sliwa, Christine E. Seidman, Michael A. Burke, Lia Crotti, Peter J. Schwartz, Bongani M. Mayosi

**Affiliations:** 1 Cardiovascular Genetics Laboratory, Hatter Institute for Cardiovascular Research in Africa, Department of Medicine, Groote Schuur Hospital and University of Cape Town, Old Groote Schuur Hospital, Groote Schuur Drive, Observatory, 7925, Cape Town, South Africa; 2 Department of Genetics, Harvard Medical School, NRB 256, 77 Ave. Louis Pasteur, Boston, MA 02115, USA; 3 Division of Cardiovascular Medicine, Brigham and Women’s Hospital, 75 Francis Street, Boston, MA 02115, USA; 4 Center for Cardiac Arrhythmias of Genetic Origin and Laboratory of Cardiovascular Genetics, IRCCS Istituto Auxologico Italiano, Milan, Italy; 5 Department of Cardiology, Fondazione IRCCS Policlinico S. Matteo, Viale Golgi 19, 27100 Pavia, Italy

## Abstract

Cardiomyopathy is an important cause of heart failure in Sub-Saharan Africa, accounting for up to 30% of adult heart failure hospitalisations. This high prevalence poses a challenge in societies without access to resources and interventions essential for disease management. Over 80 genes have been implicated as a cause of cardiomyopathy. Mutations in the phospholamban (*PLN*) gene are associated with dilated cardiomyopathy (DCM) and severe heart failure. In Africa, the prevalence of *PLN* mutations in cardiomyopathy patients is unknown. Our aim was to screen 315 patients with arrhythmogenic right ventricular cardiomyopathy (n = 111), DCM (n = 95), hypertrophic cardiomyopathy (n = 40) and peripartum cardiomyopathy (n = 69) for disease-causing *PLN* mutations by high resolution melt analysis and DNA sequencing. We detected the previously reported *PLN* c.25C > T (p.R9C) mutation in a South African family with severe autosomal dominant DCM. Haplotype analysis revealed that this mutation occurred against a different haplotype background to that of the original North American family and was therefore unlikely to have been inherited from a common ancestor. No other mutations in *PLN* were detected (mutation prevalence = 0.2%). We conclude that *PLN* is a rare cause of cardiomyopathy in African patients. The *PLN* p.R9C mutation is not well-tolerated, emphasising the importance of this gene in cardiac function.

Cardiomyopathy is defined as a myocardial disease in which the heart muscle is structurally and functionally abnormal, without hypertension, coronary artery disease, valvular disease or congenital heart disease which is sufficient to cause the observed myocardial abnormality^1^. It is a major cause of heart disease in Sub-Saharan Africa, accounting for 20–30% of adults hospitalised for acute heart failure in the region^2^. Cardiomyopathy poses a challenge in Africa because of its high prevalence in resource-poor societies, the difficulty of diagnosis, the limited access to effective interventions and the associated high mortality^3^. Cardiomyopathies can be primary (genetic, mixed or acquired) or secondary (infiltrative, toxic or inflammatory)^4^.

Cardiomyopathies are known to be caused by genes that have cytoskeletal, contractile and calcium regulatory functions^5^. One of the genes implicated in cardiomyopathy is phospholamban (*PLN*), which encodes the phospholamban protein that is involved in calcium signalling and muscle contraction. Mutations in *PLN* have been associated with dilated cardiomyopathy (DCM)^6^,^7^,^8^, hypertrophic cardiomyopathy (HCM)^9^ and recently arrhythmogenic right ventricular cardiomyopathy (ARVC) in North America and Europe^10^, but the role of *PLN* in Africans with cardiomyopathy is unknown^11^. A *PLN* founder mutation, *PLN* p.R14del, was identified in large European cohorts, including 10–15% of Dutch patients with ARVC or DCM, and was associated with high mortality and poor prognosis^10^,^12^. As this mutation arose 575–825 years ago, the possibility exists that the *PLN* p.R14del founder mutation may be present in descendants of Dutch settlers and may cause cardiomyopathy in a subset of individuals of European descent in Southern Africa^13^. The aim of this study was to determine if *PLN* is a cause of cardiomyopathy in Africans, and explore the possibility of *PLN* founder mutations common between the African population and individuals with European ancestry.

## Results

**Figure 1:**
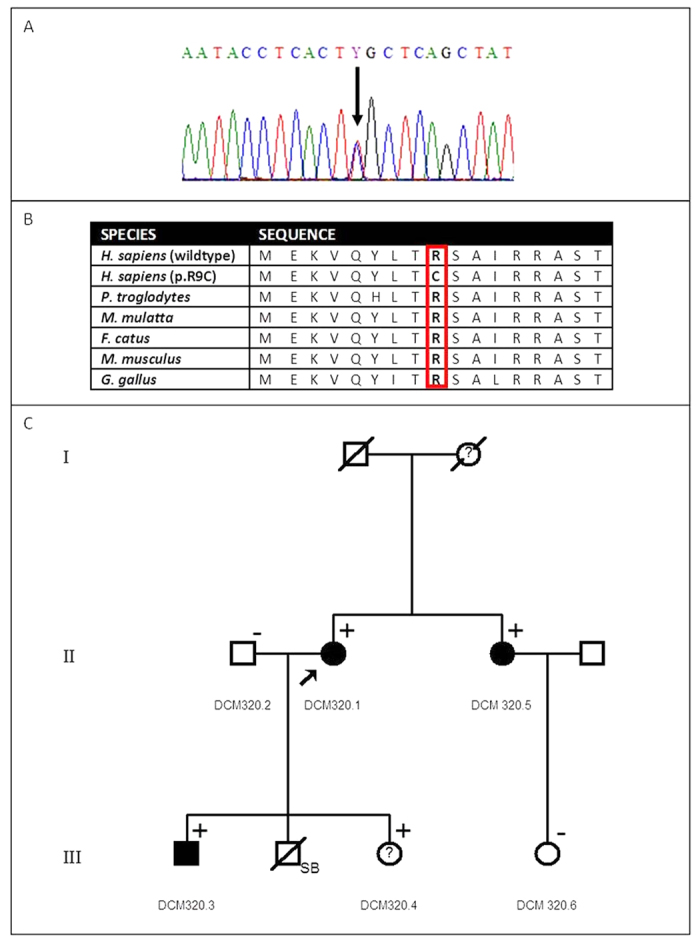
Identification of the c.25C > T (p.R9C) PLN mutation. (**A**) Electropherogram showing the c.25C > T sequence change. (**B**) Multiple species protein alignment of this sequence. (**C**) Pedigree of family DCM 320 showing variant c.25C > T and individuals with DCM. SB: Stillbirth; Gender (Square – Male; Circle – Female); Clinical Status (Black symbol – Affected; Clear symbol – Unaffected;? – Diagnosis uncertain); Mutation Status ( + – c.25C > T positive; – – c.25C > T negative).

Mutation screening of *PLN* in 315 patients with cardiomyopathy (ARVC (n = 111)^14^, DCM (n = 95)^15^, HCM (n = 40)^16^, and peripartum cardiomyopathy (PPCM) (n = 69))^17^ revealed the previously reported c.25C > T (p.R9C) mutation in a proband of European descent with severe DCM (DCM 320.1; Individual II:2)^6^. This mutation results in the alteration of a conserved amino acid (Fig. 1A,B) from an arginine (R) to a cysteine (C) and was absent in 200 ethnically matched control chromosomes. No *PLN* mutations were detected in individuals with ARVC, HCM or PPCM.

Detailed investigations into the ancestry of individual DCM 320.1 pointed to an autosomal dominant inheritance pattern within this DCM family (Fig. 1C). The proband (DCM 320.1; II:2) had a heart transplant at the age of 35 years while her younger sister (DCM 320.5; II:3) was likewise affected with DCM and required a heart transplant at the age of 39 years. The proband’s son (DCM 320.3; III:1) developed DCM at the age of 24 years and underwent a heart transplant a year later. The echocardiogram showed features of DCM with a left ventricular ejection fraction of 20%. Coronary angiography showed patent epicardial coronary arteries, and left ventriculography displayed a dilated left ventricle with poor systolic function. The proband’s daughter (DCM 320.4; III:3) was asymptomatic but the echocardiogram showed borderline dilatation of the left ventricle (left ventricular end diastolic dimension of 5.3 cm) and left ventricular ejection fraction of 47%. Further enquiry also revealed that the proband’s mother had died of a heart condition at the age of 36 years. However, we were not able to confirm whether this individual was affected with DCM.

Subsequent mutation screening of *PLN* for the available members of this family found the c.25C > T (p.R9C) mutation in the proband’s affected son (DCM 320.3; Individual III:1) and daughter (DCM 320.4; Individual III:3) as well as the proband’s affected sister (DCM 320.6;Individual II:3), reflecting segregation of the c.25C > T mutation with DCM in this family.

Bioinformatic analysis tools MutationTaster, PolyPhen-2 and Align GVGD predicted the *PLN* c.25C > T (p.R9C) mutation to be disease-causing. Further, transgenic mice bearing the *PLN* p.R9C mutation developed severe DCM and underwent premature death, confirming the pathogenicity of this mutation^6^. Bioinformatic RNA tools mfold and RNAfold predicted this variant would change mRNA secondary structure whereas the ESEfinder tool predicted that this variant would alter serine/arginine-rich splicing factor (SRSF1 and SRSF5) exonic splice enhancer recognition sites.

**Figure 2:**
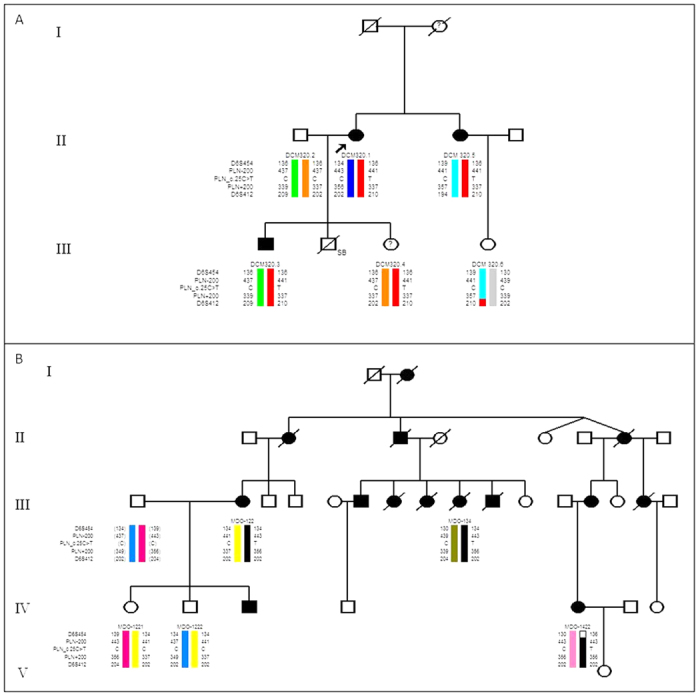
Haplotype analysis. (**A**) Pedigree of the DCM 320 family. (**B**) Pedigree of the MDO DCM family.

As the *PLN* c.25C > T mutation was previously reported in a family of European descent by Schmitt and colleagues^6^, we wanted to ascertain if these mutations arose independently or if they were inherited from a common ancestor, possibly as early as the 17^th^ century with the European colonisation of South Africa. Haplotypes were constructed using published microsatellite markers spanning 5.15 Mb across the *PLN* gene (D6S454, PLN −200 K, PLN +200 K and D6S412)^13^. In the DCM 320 family, the *PLN* c.25C > T mutation was found to be part of a disease-specific haplotype spanning this 5.15 Mb region (Fig. 2A). The *PLN* c.25C > T mutation was found to be part of a different haplotype spanning 1.85 Mb in the MDO DCM family described by Schmitt *et al.*^6^ (Fig. 2B).

## Discussion

In the initial report of the *PLN* c.25C > T (p.R9C) mutation by Schmitt and colleagues^6^, the mutation was associated with an autosomal dominant inheritance pattern in DCM which was characterised by increased cardiac chamber dimensions, decreased contractile function at 20–30 years of age and progression to heart failure within 5–10 years after symptom onset^6^, a phenotype remarkably similar to that observed in the DCM 320 family. Similarly, in the recent report by Truszkowska and others, the *PLN* p.R9C mutation was detected in an individual with acute onset of DCM at the age of 21 years, leading to heart transplantation at 22 years of age^18^.

Functional analysis of the effect of the *PLN* p.R9C mutation on the protein and its role in the development of DCM revealed significant consequences of this mutation. A transgenic mouse model harbouring the p.R9C mutation was shown to develop DCM^6^. Further evidence has suggested that *PLN* p.R9C results in decreased responsiveness to β-adrenergic stimulation secondary to reduced phosphorylation by protein kinase A^6^,^19^,^20^. This results in altered calcium kinetics and aberrant contractility. The presence of severe DCM in this family with *PLN* p.R9C reinforces the importance of this mutation in the pathogenesis of DCM.

We have established that the *PLN* c.25C > T mutation forms part of a disease-specific haplotype spanning 5.15 Mb across *PLN* in the DCM 320 family that differed from the 1.85 Mb haplotype observed in the MDO DCM family described by Schmitt *et al.*^6^. It is therefore unlikely that this mutation was inherited from a common ancestor. By contrast, the *PLN* p.R14del mutation, has been identified as a founder mutation in a large Dutch cohort of patients with ARVC, as well patients from Germany and Spain^13^,^21^. A mouse model with cardiac-specific overexpression of this mutation presented with DCM associated with arrhythmias, cardiac fibrosis and premature death^8^.

Although a total of 315 South African cardiomyopathy patients (ARVC, DCM, HCM, and PPCM) were screened in this study, only one disease-causing mutation was found in *PLN* (frequency of 0.2%) illustrating that *PLN* is not a common cause of cardiomyopathy in South Africa. The failure to detect *PLN* mutations in patients with PPCM is consistent with the findings of others^22^. To date, there are six *PLN* mutations (c.25C > T (p.R9C)^6^,^18^, c.26G > T (p.R9L)^23^,^24^, c.26G > A (p.R9H)^24^, c.40_42delAGA (p.R14del)^8^,^10^,^13^,^25^,^26^, c.73C > T (p.R25C)^27^ and c.116T > G (p.L39X)^7^) that are associated with DCM, one with ARVC (c.40_42delAGA (p.R14del)^10^) and three with HCM (c.116T > G (p.L39X)^9^,^28^, c.1-77A > G^29^ and c.1-42C > G^30^) (Table 1).

While the majority of these *PLN* mutations have been associated with severe cardiomyopathy, only *PLN* mutations associated with DCM meet the strict criteria to be called definitively pathogenic. These are nonsynonymous variants that alter a highly conserved amino acid residue across species, are absent from large numbers of healthy controls, demonstrate statistically significant co-segregation with affected members of a family, and are identified by an unbiased genetic approach. Important additional evidence comes from (1) the recapitulation of disease in animals engineered to express the variant, as exists for the PLN p.R9C mutation^6^ or (2) the same mutation is identified in unrelated individuals with the disease phenotype, as we have demonstrated herein for the *PLN* p.R9C mutation.

As far as we are aware, the three PLN mutations detected in patients with HCM are disease-associated and cannot be classified as definitely pathogenic for several reasons. First, the authors who reported the *PLN* c.116T > G (p.L39X) mutation in HCM provide evidence indicating the association of this mutation with HCM in a single proband^31^. There is no apparent familial segregation of this mutation with disease. The authors also provide no functional evidence demonstrating the role of this mutation in disease pathogenesis. Other authors provide evidence that this mutation is associated with disease in a single proband with HCM. The proband’s daughter carried the mutation but was unaffected with HCM, while the 3 year old granddaughter was affected with this disease^28^. The authors suggest that this may be due to incomplete penetrance associated with this mutation, but the fact that the affected granddaughter is so young calls this explanation into question. Also, no functional evidence is provided for the role of this mutation in DCM pathogenesis. Second, the *PLN* c.1-77A > G has been associated with HCM in a single proband^29^. No evidence for familial segregation or functional evidence suggesting a role of this mutation in HCM pathogenesis is provided. Finally, Medin *et al.* report the *PLN* c.1-42C > G variant as associated with disease in a family with HCM^32^. One of the proband’s sons was unaffected with HCM even though he carried the mutation, which may be explained by incomplete penetrance. However, no functional evidence suggesting the role of this variant in DCM pathogenesis is provided. For these reason, we classify these three variants as HCM-associated but not definitely pathogenic (Table 1).

In conclusion, we have identified the previously characterised c.25C > T *PLN* mutation that segregates with severe DCM in a South African kindred following the screening of 315 patients with different types of cardiomyopathy. No *PLN* mutations were identified in subjects with ARVC, HCM or PPCM. *PLN* mutations are a rare cause of cardiomyopathy in South Africans which should be added to screening panel for cardiomyopathy in the country.

## Methods

The methods were carried out in accordance with the approved guidelines of the University of Cape Town Human Research Ethics Committee, and they are presented in terms of samples, genetic screening, and bioinformatic analysis, and microsatellite analysis below.

### Samples

South African patients with ARVC (n = 111; 46% of European descent), DCM (n = 95; 8% of European descent), HCM (n = 40; 33% of European descent) and PPCM (n = 69; 10% of European descent) were referred to the Cardiac Clinic in Groote Schuur Hospital, Cape Town for genetic evaluation. ARVC cases were enrolled in the Arrhythmogenic Right Ventricular Cardiomyopathy Registry of South Africa. The diagnosis of ARVC was made according to the modified criteria set by the Task Force of the Working Group on Myocardial and Pericardial Diseases of the European Society of Cardiology^33^. DCM, PPCM and HCM patients were diagnosed on the basis of the definitions of the European Society of Cardiology^1^. The details on methods of clinical evaluation, demographics and clinical parameters of patients enrolled in this study have been described previously^14^,^15^,^16^,^17^.

Blood samples were collected for molecular genetic testing. The population controls were anonymous blood donors from the Western Province Blood Transfusion Service who provided blood samples for DNA isolation. The study was approved by the Human Research Ethics Committee of the University of Cape Town, and informed consent was obtained from all participants. All ARVC cases included in this study had previously been screened for mutations in the desmosomal genes (*DSP*, *PKP2*, *DSC2*, *DSG2* and *JUP*) known to cause ARVC but were found not to harbour any pathogenic mutations. In our screen we included 315 ARVC, DCM, HCM and PPCM cases with unidentified pathogenic mutations.

### Genetic screening

Mutation screening of *PLN* included 111 ARVC, 95 DCM, 40 HCM, and 69 PPCM patients and was performed by high resolution melt analysis (HRM) and Sanger sequencing using the following primers: Forward – 5′-CCAGGCTACCTAAAAGAAGAC-3′; Reverse – 5′-TTCCTGTCTGCATGGGATG-3′. HRM is proven mutation screening method with a high sensitivity and specificity^34^. HRM reactions were prepared using 0.5U GoTaq^®^ Flexi DNA Polymerase (Promega, Madison, WI, USA), 5X Colorless GoTaq^®^ Flexi Buffer (Promega), 3 mM (final reaction concentration) MgCl_2_ (Promega), 0.8 μM dNTPs (final reaction concentration) (Bioline, London, United Kingdom), EvaGreen dye (Biotium, Hayward, CA, USA) and 0.4 μM of each primer (final reaction concentration). HRM reactions were conducted using the RotorGene 6000 (Corbett Life Sciences – Qiagen, Venlo, Limber, Netherlands) and conditions were 95 °C for 10 minutes, 50 cycles of 95 °C for 5 seconds, 55 °C for 10 seconds and 72 °C for 10 seconds, and a high resolution melt from 72 °C to 95 °C with 0.1 °C increases in temperature. Samples displaying changes in HRM profiles relative to controls were earmarked for purification and sequencing. HRM products were purified using *Exonuclease I* (New England Biolabs, Ipswich, MA, USA) and *FastAP*^*TM*^ *Thermosensitive Alkaline Phosphatase* (Promega) using a Mastercycler^®^ pro thermal cycler (Eppendorf, Hamburg, Germany); conditions were 37 °C for 1 hour and 75 °C for 15 minutes. Sequencing reactions were prepared using the BigDye^®^ Direct Cycle Sequencing Kit (Applied Biosystems – Life Technologies, Carlsbad, CA, USA). Reaction conditions were 96 °C for 5 minutes and 25 cycles of 96 °C for 30 seconds, 50 °C for 15 seconds and 60 °C for 4 minutes. Sequencing products were analysed using capillary electrophoresis with the ABI PRISM^®^ 3130 × l Genetic Analyser (Applied Biosystems) at the DNA Sequencing Unit (Department of Genetics, Stellenbosch University, Cape Town). The prevalence of variants of interest in the control population was determined by screening 200 ethnically matched control chromosomes by high resolution melt analysis and Sanger sequencing.

### Bioinformatic analysis

Bioinformatic analysis of the *PLN* variants of interest was conducted using various bioinformatic tools [mfold (http://mfold.rna.albany.edu/?q=mfold/RNA-Folding-Form), RNAfold (http://rna.tbi.univie.ac.at/cgi-bin/RNAfold.cgi), ESEFinder (http://rulai.cshl.edu/cgi-bin/tools/ESE3/esefinder.cgi?process=home), PolyPhen-2 (http://genetics.bwh.harvard.edu/pph2/), SIFT (http://sift.jcvi.org/www/SIFT_seq_submit2.html), Align GVGD (http://agvgd.iarc.fr/agvgd_input.php) and MutationTaster (http://www.mutationtaster.org/)] to assess the impact of the variants on *PLN* structure and function at the mRNA and protein levels.

### Microsatellite analysis

MDO DCM DNA samples for microsatellite analysis were obtained from Professor Christine Seidman (Department of Genetics, Harvard Medical School, Boston, MA). The D6S454^13^, *PLN* −200 K^13^, *PLN* +200 K^13^ and D6S412^13^ microsatellite markers were used to construct haplotypes for *PLN*. PCR products were run on a 2% agarose gel for verification of reaction success and product size and specificity. These products were then analysed using capillary electrophoresis with the ABI PRISM^®^ 3130 × l Genetic Analyser (Applied Biosystems, Foster City, CA, USA) at the Division of Human Genetics, University of Cape Town. Results were analysed using GeneMapper^®^ v4.1 software (Applied Biosystems) and haplotypes were constructed using Cyrillic v2.0 (Cyrillic Software, United Kingdom).

## Additional Information

**How to cite this article**: Fish, M. *et al.* Mutation analysis of the phospholamban gene in 315 South Africans with dilated, hypertrophic, peripartum and arrhythmogenic right ventricular cardiomyopathies. *Sci. Rep.* **6**, 22235; doi: 10.1038/srep22235 (2016).

## Figures and Tables

**Table 1:** Known *PLN* variants associated with cardiomyopathy.

Variant	Disease	No. of probands carrying variant	Frequency in control chromosomes	Definitely Pathogenic	References
c.25C > T (p.R9C)	DCM	2	0/200; 0/400	Yes	6,18
c.26G > T (p.R9L)	DCM	1	0/1000	Unknown	23,24,35,36
c.26G > A (p.R9H)	DCM	1	0/1000	Unknown	24,36
c.40_42delAGA (p.R14del)	DCM, ARVC	39 DCM, 13 ARVC^10^; 1 DCM^8^; 1 DCM^25^; 101 DCM and ARVC^13^; 1 DCM^26^; 1 DCM^24^; 1 ARVC^21^	1/946^10^; 0/1316^8^;0/200^25^;6/16434^13^; 0/1000^24^	Yes	8,10,13,21,24, 25, 26,37
c.73C > T (p.R25C)	DCM	1	NA	Unknown	27
c.116T > G (p.L39X)	DCM, HCM	2 DCM^7^; 1 HCM^28^; 1 HCM^9^	0/500^7^; 0/600^28^; 0/200^9^	Yes (DCM) and Unknown (HCM)	7,9,28
c.1-77A > G	HCM	1	0/592	Unknown	29
c.1-42C > G	HCM	1	0/100	Unknown	30
