# Corrigendum: Mutation analysis of the phospholamban gene in 315 South Africans with dilated, hypertrophic, peripartum and arrhythmogenic right ventricular cardiomyopathies

**DOI:** 10.1038/srep25863

**Published:** 2016-05-18

**Authors:** Maryam Fish, Gasnat Shaboodien, Sarah Kraus, Karen Sliwa, Christine E. Seidman, Michael A. Burke, Lia Crotti, Peter J. Schwartz, Bongani M. Mayosi

Scientific Reports 6: Article number: 2223510.1038/srep22235; published online: 02262016; updated: 05182016

In this Article, Lia Crotti is incorrectly affiliated with ‘Department of Cardiology, Fondazione IRCCS Policlinico S. Matteo, Viale Golgi 19, 27100 Pavia, Italy’. Furthermore, an additional affiliation was omitted. The correct affiliations are listed below:

Center for Cardiac Arrhythmias of Genetic Origin and Laboratory of Cardiovascular Genetics, IRCCS Istituto Auxologico Italiano, Milan, Italy

Department of Molecular Medicine, University of Pavia, Italy

Peter J. Schwartz is incorrectly affiliated with ‘Cardiovascular Genetics Laboratory, Hatter Institute for Cardiovascular Research in Africa, Department of Medicine, Groote Schuur Hospital and University of Cape Town, Old Groote Schuur Hospital, Groote Schuur Drive, Observatory, 7925, Cape Town, South Africa’ and ‘Department of Cardiology, Fondazione IRCCS Policlinico S. Matteo, Viale Golgi 19, 27100 Pavia, Italy. The correct affiliation is listed below:

Center for Cardiac Arrhythmias of Genetic Origin and Laboratory of Cardiovascular Genetics, IRCCS Istituto Auxologico Italiano, Milan, Italy

